# Massively Parallel Amplicon Sequencing Reveals Isotype-Specific Variability of Antimicrobial Peptide Transcripts in *Mytilus galloprovincialis*

**DOI:** 10.1371/journal.pone.0026680

**Published:** 2011-11-07

**Authors:** Umberto Rosani, Laura Varotto, Alberta Rossi, Philippe Roch, Beatriz Novoa, Antonio Figueras, Alberto Pallavicini, Paola Venier

**Affiliations:** 1 Department of Biology, University of Padua, Padova, Italy; 2 Ecologie des Systèmes Marins et Côtiers, CNRS-IRD-University of Montpellier 2, Montpellier, France; 3 Instituto de Investigaciones Marinas, CSIC, Vigo, Spain; 4 Department of Life Sciences, University of Trieste, Trieste, Italy; The Centre for Research and Technology, Greece

## Abstract

**Background:**

Effective innate responses against potential pathogens are essential in the living world and possibly contributed to the evolutionary success of invertebrates. Taken together, antimicrobial peptide (AMP) precursors of defensin, mytilin, myticin and mytimycin can represent about 40% of the hemocyte transcriptome in mussels injected with viral-like and bacterial preparations, and unique profiles of myticin C variants are expressed in single mussels. Based on amplicon pyrosequencing, we have ascertained and compared the natural and *Vibrio*-induced diversity of AMP transcripts in mussel hemocytes from three European regions.

**Methodology/Principal Findings:**

Hemolymph was collected from mussels farmed in the coastal regions of Palavas (France), Vigo (Spain) and Venice (Italy). To represent the AMP families known in *M. galloprovincialis*, nine transcript sequences have been selected, amplified from hemocyte RNA and subjected to pyrosequencing. Hemolymph from farmed (offshore) and wild (lagoon) Venice mussels, both injected with 10^7^ *Vibrio* cells, were similarly processed. Amplicon pyrosequencing emphasized the AMP transcript diversity, with Single Nucleotide Changes (SNC) minimal for mytilin B/C and maximal for arthropod-like defensin and myticin C. Ratio of non-synonymous vs. synonymous changes also greatly differed between AMP isotypes. Overall, each amplicon revealed similar levels of nucleotidic variation across geographical regions, with two main sequence patterns confirmed for mytimycin and no substantial changes after immunostimulation.

**Conclusions/Significance:**

Barcoding and bidirectional pyrosequencing allowed us to map and compare the transcript diversity of known mussel AMPs. Though most of the genuine cds variation was common to the analyzed samples we could estimate from 9 to 106 peptide variants in hemolymph pools representing 100 mussels, depending on the AMP isoform and sampling site. In this study, no prevailing SNC patterns related to geographical origin or *Vibrio* injection emerged. Whether or not the contact with potential pathogens can increase the amount of AMP transcript variants in mussels requires additional study.

## Introduction

*Mytilus* species (Phylum Mollusca, Class Bivalvia) are intertidal filter-feeders distributed worldwide, anchored to hard substrates in dense communities and widely used as bio-sensors of coastal pollution. Mussel populations of the northern and southern hemisphere probably separated 0.54–1.31 million years ago, far after the trans-Arctic expansion towards North America, and before the divergence between the Atlantic and Mediterranean ecotypes [1]. *M. galloprovincialis* hybridizes with *M. edulis* in southwest England and the Mediterranean mussel is now reported in Eastern Asia, California, Chile and Western Australia [1]–[2].

At different latitudes, mussels face tidal and seasonal fluctuations, changeable pollutant loads and also the surrounding bioma with behavioral changes [3], metabolic adjustments [4] and a variety of defense reactions [5]–[7]. With the exception of a few metazoan parasites which also somewhat affect the *Mytilus* species [8]–[9], mussels seem refractory to diseases and could instead influence the prevalence of pathogens such as *Perkinsus* spp. and *Betanodavirus* (Nodaviridae) in other bivalves and fishes, respectively [10]. Like other invertebrates, bivalve molluscs rely on ancient and rapid defenses to fight potential pathogens, and gene-encoded antimicrobial peptides (AMPs) are major humoral components of their immune system.

Host defense peptides are present in virtually all living organisms, with more than 30 AMPs expressed in humans and about 200 peptides identified in insects (approximately 1500 molecules very diverse in sequence and secondary structures are reported in specific databases). [11]–[13]. Among other structural features, a conserved γ-core motif originated from the bidirectional orientation of specific aminoacid residues including an invariant cysteine array has indicated the evolutionary relatedness of cysteine-stabilized α-β (CS-αβ) AMPs, kinocidins, invertebrate toxins and snake venoms: such unifying structure provides an interesting hypothesis for context-specific action modes, from the perturbation of negatively charged cell membranes and ion channels to the immunoregulatory functions [14].

In the continuous fight with competitors, predators and pathogens, the evolutionary diversification of AMP types and gene families likely occurred through events of gene duplication, shuffling of functional elements and selection for variation at positions adjacent, or integral to, the conserved structural motifs [13], [15]. In *Crassostrea gigas*, combined mechanisms of sequence diversification (e.g. recombination, parallel homoplasic mutations, indel events) and directional selection have been suggested to explain the remarkable gene multiplicity and variable copy number of defensins and proline-rich peptides, whereas the marked transcript diversity of Cg-bpi, a bactericidal permeability protein, has been mainly referred to the allelic polymorphism of one single gene [16]. Microsatellite-mediated mosaics of sequence elements, low-transcription fidelity and transcript editing support the evidence of about 50 polymorphic genes, and an extraordinary diverse set of Sp185/333 proteins expressed in response to pathogens by the purple sea urchin [17]–[18]. Worthy of note, the copy number polymorphism of α and β defensin genes with proportional peptide levels in neutrophylic granulocytes, has been related to the individual risk of infection in humans [19]–[20].

Tens of different AMPs or AMP families have been discovered in marine invertebrates [21]. In the mussels *M. galloprovincialis* and *M. edulis*, four different groups of CS- αβ AMPs with multiple isoforms have been discovered and classified according to their primary sequence and secondary structure: defensins reported as MGD1 and MGD2, mytilin A, B, C, D and E, myticin A, B and C, mytimycin, the only strictly antifungal peptide with an EF-hand like domain [22]–[25]. These AMPs share small size (3.7–4.5 kDa, except mytimycin of 6.2 kDa), positive charge and amphiphilic behavior. Their precursors (pre-pro-peptides) consist of an N-terminal signal peptide, a central mature peptide and a C-terminal extension. Each family is characterized by a cysteine array of 8 (12 in mytimycin) cysteines engaged in intramolecular disulfide bonds.

A broad spectrum of activity, often complementary and not strictly antibacterial, was reported for the mussel defensins, mytilins and myticins [26]–[27] whereas mytimycin, a 6.2 kDa peptide isolated from normal and immunostimulated mussels, selectively inhibited *Neurospora* and *Fusarium* growth [22]. Whether purified in sufficient amounts from cellular fractions or obtained in stable conformations by chemical synthesis or recombinant system, pure peptides are essential to investigate the antibiotic power of the different mussel AMPs.

*In situ* hybridization and immunolocalization assays performed on mussel hemocytes demonstrated a partially overlapping expression of defensins and mytilins [28]. AMP expression and stored peptides have been observed in several tissues and developmental stages [28]–[29] to indicate that cells other than hemocytes can produce and release AMPs, a phenomenon well known from frog skin [30] and the male reproductive system of rats [31]. Overall, mussel AMPs display rather complex expression patterns, dependent on developmental stage, seasonality and immunostimulation [32]–[34]. In *M. galloprovincialis*, massive EST sequencing confirmed the abundance and transcript diversity of AMPs and other key players of the innate immunity [35]. The AMP precursors represented 26–43% of the hemocyte transcripts in mussels injected with viral-like and bacterial preparations; in particular, 74 precursor and 25 mature peptide variants of myticin C were detected in a sample of only 100 mussels, with unique profiles of transcript variants in single mussels and less common alleles differing at single nucleotide positions from the two most common ones [29], [36].

The myticin C variation is also remarkable compared to mytilin B, as one mussel can produce 2–10 different mytilin B transcripts but silent substitutions restrict the peptide variants to only a few [37]. In spite of the abundance of other AMPs, just one singleton plus 4 similar sequences denote mytimycin in Mytibase, interactive catalogue including 18788 expressed sequence tags (ESTs) of *M. galloprovincialis* [25]. Sequencing and Southern blot data indicate one gene copy per genome for defensin MGD2, mytilin B and myticin C [38], [36]. Two gene copies or allelic polymorphism could explain the simultaneous presence of two length variants of the mytimycin gene per mussel [39]. The gene copy number of the mussel AMPs need verification since partial gene sequences covering the coding sequence (cds) are only available for MGD1, mytilin B, myticin C and mytimycin [29], [35]–[39]. Two 3D structures have been established by NMR spectrometry, defensin MGD1 [40] and mytilin B [41].

Thus far no *Mytilus* genome has been sequenced and, compared to better known model organisms, a limited number of genes have been investigated: for instance those concerning defensin and mytilins [38], heat shock proteins [42], metallothioneins [43] and apoptotic caspases [44]. Also gene-centered studies take advantage of the massive production of ESTs which currently contributes to the identification of molecules and pathways underlying the mussel response to various natural and experimental conditions [45]–[48]. Among the 67,726 ESTs and 4680 aminoacid sequences publicly available for the *Mytilus* genus, about 29 and 32%, respectively, refer to *M. galloprovincialis* (www.ncbi.nlm.nih.gov, June 2011). Recently, new ESTs have been produced from the digestive gland, foot, gill and mantle of *M. galloprovincialis* by advanced sequencing [49]. The so called ‘pyrosequencing’ was the first alternative to the use of chain-terminating inhibitors [50] and it has radically increased the sequencing power as well as the resolution of low-abundance variants [51]–[53]. Adequate read coverage can assure reliable quantification of single nucleotide changes (SNC) when seeking critical mutations or sequence polymorphisms.

Based on 454 pyrosequencing, we have thoroughly studied the sequence diversity of 9 different AMP precursors expressed in hemocytes of mussels (*M. galloprovincialis*) farmed in three European regions. Similarly, we have analyzed and compared mussels farmed offshore or living inside the Venice Lagoon (Italy), before and after injection with live *Vibrio* cells.

## Results

In Table 1 we summarized the main features of nine CS- αβ AMPs expressed in *M. galloprovincialis*.

**Table 1:** Features of the selected mussel AMPs.

AMP	Precursor length (aa)	Mature peptide length (aa)	Cysteine array	Weight (kDa)	Isoelectric point (pH)	Hydrophobicity ratio (%)
**MytA**	96	40	8	4.5	9.1	35
**MytB**	96	40	8	4.6	9.2	30
**MytC**	100	40	8	4.4	8.8	35
**MytlB**	103	34	8	4.0	9.7	27
**MytlC**	100	34	8	4.2	9.9	24
**MytlD**	97	34	8	3.9	10.4	32
**MytM**	152	54	12	6.4	8.4	26
**MGD1**	82	39	8	4.4	9.4	36
**MGDt**	61	38	8	4.4	9.3	45

MGDt lacks of C-terminal extension. Table data are calculated from Mytibase ESTs. See also AF162334.1, AF162335.1, AF162336.1, AF162337.1, EU810204.1 and EU927448.1 at NCBI.

Following appropriate primer design, we amplified the related transcript sequences from hemolymph pools representing groups of 100 mussels farmed in south France (Pa), northwest Spain (Vi) and northeast Italy (Ve) or native from the industrial canals of the Lagoon of Venice (Ve nc). In addition, groups of 40 offshore-farmed (Ve ft) and lagoon-native (Ve nt) mussels were injected with 10^7^ live *Vibrio splendidus* cells and similarly processed (Table 2). The resulting 78 PCR products (13 amplicons×6 samples) were then purified, quantified, diluted to the appropriate concentration (∼7*10^9^ molecules/µl) to compose two equimolecular pools for the emulsion PCR (cDNA concentration was 2.3 and 2.5 ng/µl, respectively) and bidirectional sequencing.

**Table 2:** Description of the mussel haemolymph samples processed for 454 pyrosequencing.

Sample ID	Origin	No. of mussels	Status	Treatment
Pa	Palavas, F	100	farmed	untreated
Vi	Ria de Vigo, S	100	farmed	untreated
Ve	Offshore Venice, I	100	farmed	untreated
Ve nc	Venice Lagoon, I	100	native	untreated
Ve ft	Offshore Venice, I	40	farmed	*Vibrio* injection
Ve nt	Venice Lagoon, I	40	native	*Vibrio* injection

Overall, massively parallel sequencing produced 359,867 output reads and more than 73 Mbases with good quality scores, for a total of 304,621 trimmed reads with 226 bp average length. We discarded about 15% of reads per sample (88% of them shorter than 70 bp) which possibly originated in the PCR amplification or sequencing reaction (short sequences are not expected to bias the amplicon coverage nor the accuracy of SNCs detection).

**Figure 1:**
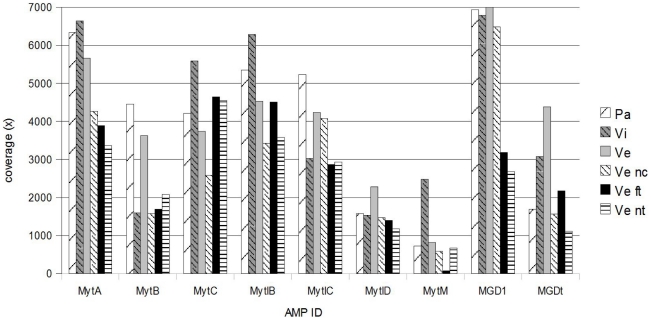
Average base coverage (x) per AMP and sample. Coverage is calculated as total sequenced base divided by the length of the amplified transcript.

Total or partial overlapping of the forward and reverse reads allowed the complete coverage of the 13 reference sequences. Hence, 97.5% of the good quality reads correctly mapped against the 9 selected AMPs and could be attributed to the 6 original samples. With the exception of MytM in the sample Ve ft, at least 1034 reads mapped on each AMP precursor transcript (range 1034–10814, File S3). Figure 1 shows relevant differences in the average base coverage calculated per AMP precursor transcript in each sample (3378×, the average read depth per AMP).

Separately for each AMP and sample, we subsequently grouped the reads having the same length and 100% identity in two cluster types: (1) equal to the original transcript and (2) with at least 1 SNC. The last ones were employed for SNC detection and related analysis. Only the SNCs covered 30×, and representing at least 3% of the reads mapping a given AMP, were considered genuine and counted per AMP precursor (Table 3). Based on the SNC counts, the average value of SNC per base calculated for the coding sequence was 0.18. Considering all AMPs together, as much as 134 SNCs were common to the 6 samples and represented 86% of the genuine cds variation (File S4). The SNC frequency values (cds) indicated MytC and MGD1 as the most polymorphic AMP transcripts, opposite to those of mytilins B and C. The fraction of non-synonymous changes ranged from null (MytlC) to 25 (MytC) whereas the related ω values highlighted the opposite cases of MytlC (null) and MytlD (9–15).

**Table 3:** Number of SNCs detected in the whole AMP transcript precursor and cds, related frequencies, non-synonymous changes and ω values.

AMP precursor	Sample	SNCs (total)	SNCs (cds)	Frequency (SNC/base)	Freq/AMP (Mean ± SD)	ns SNCs*	ω**
**MytA**	**Pa**	38	25	0.26	0.25±0.04	10	0.7
	**Vi**	36	20	0.21		8	0.7
	**Ve**	41	28	0.29		12	0.8
	**Ve nc**	38	23	0.24		10	0.8
	**Ve ft**	30	19	0.20		10	1.1
	**Ve nt**	39	26	0.27		12	0.9
**MytB**	**Pa**	21	17	0.18	0.20±0.05	12	2.4
	**Vi**	40	29	0.30		15	1.1
	**Ve**	22	17	0.18		11	1.8
	**Ve nc**	30	19	0.20		11	1.4
	**Ve ft**	27	18	0.19		12	2.0
	**Ve nt**	19	14	0.14		7	1.0
**MytC**	**Pa**	33	27	0.27	0.26±0.04	19	2.4
	**Vi**	41	33	0.33		25	3.1
	**Ve**	31	23	0.23		17	2.8
	**Ve nc**	34	26	0.26		17	1.9
	**Ve ft**	32	24	0.24		19	3.8
	**Ve nt**	27	22	0.22		15	2.1
**MytlB**	**Pa**	11	5	0.05	0.09±0.03	1	0.3
	**Vi**	17	10	0.10		5	1.0
	**Ve**	16	9	0.09		3	0.5
	**Ve nc**	23	13	0.13		9	2.3
	**Ve ft**	15	10	0.10		5	1.0
	**Ve nt**	16	9	0.09		4	0.8
**MytlC**	**Pa**	8	1	0.01	0.01±0.01	0	0.0
	**Vi**	8	1	0.01		0	0.0
	**Ve**	11	2	0.02		0	0.0
	**Ve nc**	7	1	0.01		0	0.0
	**Ve ft**	6	1	0.01		0	0.0
	**Ve nt**	8	2	0.02		0	0.0
**MytlD**	**Pa**	23	16	0.17	0.15±0.04	15	15.0
	**Vi**	25	20	0.22		18	9.0
	**Ve**	21	12	0.13		11	11.0
	**Ve nc**	19	11	0.12		11	[11]
	**Ve ft**	18	13	0.14		13	[13]
	**Ve nt**	19	10	0.11		9	9.0
**MytM**	**Pa**	28	28	0.20	0.21±0.01	19	2.1
	**Vi**	29	29	0.21		21	2.6
	**Ve**	31	31	0.22		23	2.9
	**Ve nc**	30	30	0.21		22	2.8
	**Ve ft**	29	29	0.21		21	2.6
	**Ve nt**	28	28	0.2		20	2.5
**MGD1**	**Pa**	28	21	0.26	0.28±0.02	14	2.0
	**Vi**	29	23	0.28		15	1.9
	**Ve**	28	21	0.26		14	2.0
	**Ve nc**	31	24	0.30		15	1.7
	**Ve ft**	28	22	0.27		15	2.1
	**Ve nt**	30	23	0.28		15	1.9
**MGDt**	**Pa**	43	12	0.19	0.18±0.02	11	11.0
	**Vi**	39	13	0.21		10	3.3
	**Ve**	40	12	0.19		10	5.0
	**Ve nc**	41	10	0.16		9	9.0
	**Ve ft**	35	12	0.19		10	5.0
	**Ve nt**	36	10	0.16		9	9.0

Frequency values, ns SNCs and ω values refer to the cds; * ns SNCs, non-synonymous changes;

**ω, ratio between non-synonymous and synonymous SNCs (in brackets, cases without synonymous substitutions).

**Figure 2:**
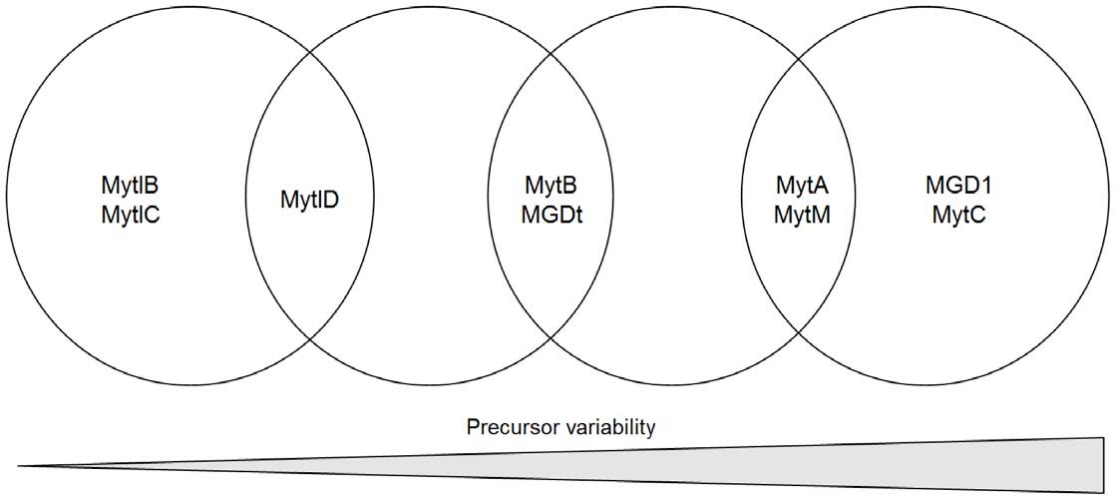
AMP precursors grouped on the basis of their variability. Grouping based on HSD test, α = 0.05; sample-specific and averaged diversity levels are reported in Table 3.

All SNC frequency values were used to test statistically the sequence diversity of the 9 selected AMPs (one-way ANOVA, α = 0.001, followed by Tukey's Honestly Significant Difference test, α = 0.05). The null hypothesis (equal SNC frequency between AMPs) was rejected and, according to the Tukey's HSD test we classified the 9 cases from the least changeable mytilins, to the most polymorphic myticins and defensins (Figure 2).

**Figure 3:**
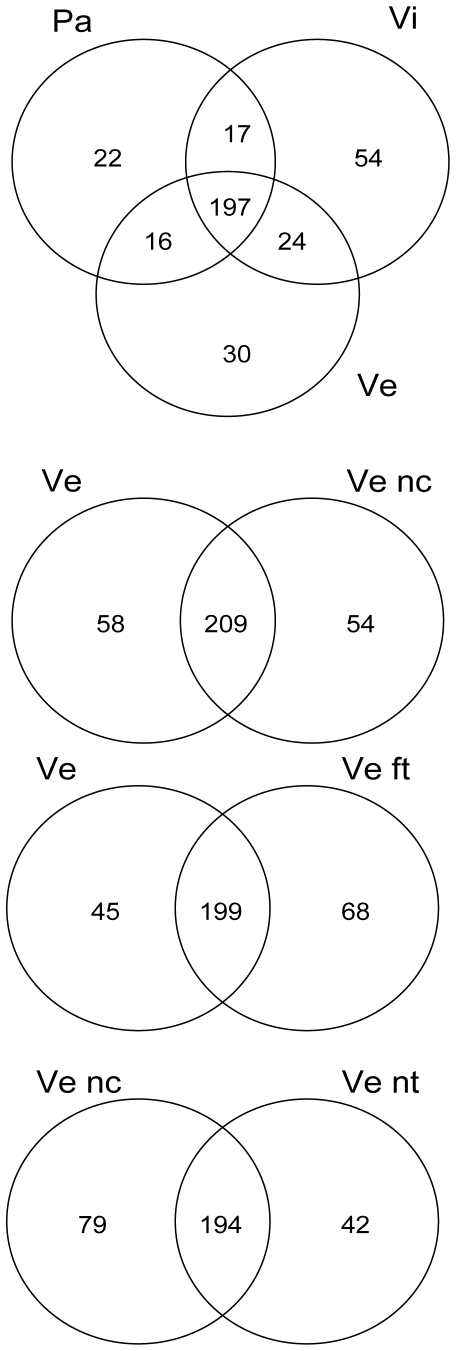
Common and exclusive SNCs in the Pa, Vi and Ve samples and paired samples from native and immunostimulated mussels.

Common and exclusive SNCs are reported in Figure 3 per geographical region: the common changes represent the majority, with 68, 74 and 78% in the Vi, Ve and Pa samples, respectively. Similar percentages of common SNCs were found in mussels farmed offshore or living wild in the industrial canals of the Venice lagoon (Ve versus Ve nc), injected or not with 10^7^ live *V. splendidus* (Ve versus Ve ft, Ve nc versus Ve nt). Details on the immune stimulation and related host response are reported elsewhere [34], [35].

Finally, we analyzed the nucleotidic and aminoacidic substitution patterns of each AMP precursor, separately in the samples from Palavas, Vigo and Venice. The percentage of read clusters differing at least 1 SNC ranged from 56% (MytlD) to 100% (MytB and MGDt). The read clusters covered at least 3× were virtually translated into amino acids, and redundancy due to silent substitutions was removed. As reported in Table 4 we could estimate a number of AMP transcript variants ranging from 9 (Pa MytM) to nearly 100 (Ve MytB, Vi MGD1, Ve MGD1 and Ve MGDt).

**4:** AMP transcript variants and expected number of non-redundant peptides per geographical region.

AMP ID	Sample	Total read clusters*	Fraction with at least 1 SNC (%)	Non-redundant peptides**
**MytA**	Pa	300	83	50
	Vi	684	90	68
	Ve	486	86	51
**MytB**	Pa	763	100	95
	Vi	280	100	63
	Ve	654	100	106
**MytC**	Pa	844	97	41
	Vi	777	100	93
	Ve	484	99	59
**MytlB**	Pa	206	70	18
	Vi	338	90	40
	Ve	190	88	28
**MytlC**	Pa	240	68	37
	Vi	138	83	23
	Ve	177	79	32
**MytlD**	Pa	61	64	17
	Vi	59	69	17
	Ve	61	56	14
**MytM**	Pa	31	94	9
	Vi	206	97	49
	Ve	32	94	12
**MGD1**	Pa	410	99	85
	Vi	557	99	100
	Ve	686	89	101
**MGDt**	Pa	204	100	45
	Vi	729	100	84
	Ve	793	100	103

*Total cluster number refers to reads differing in length and/or sequence.

**Virtual number of peptides differing at least for one residue.

The facts emerging from 454 pyrosequencing of the Mytimycin and Myticin C amplicons are reported here below in more detail as instructive examples.

The two gene sequences publicly available for the MytM of *M. galloprovincialis* (FJ804479.1, FJ804478.1) denote 3 exons and 2 introns, and differ only in the length of intron 2; the short and long version of it occurring simultaneously in single mussels [39]. The amplicon designed in the present work covered 429/456 bp, i.e. 94% of the cds.

The MytM pyrosequencing yielded 6645 aligned reads (33, 30 and 35% of them differing from the reference sequence in the Pa, Vi and Ve samples, respectively).

A nucleotide change in position 58 (Thymine in the place of Cytosine) was detected in all reads and was not considered as SNC because it could represent an error occurred during Sanger sequencing of the original Mytibase singleton (MGC05878, File S4 in Supplementary Materials). The AVA software (Roche Life Sciences) grouped the Pa, Vi and Ve MytM reads in 31, 206 and 32 clusters or singletons (27, 199 and 31 of high quality), respectively. In total, 5690 high quality sequences (86%) were translated in amino acids and produced 9–49 expected peptide variants (Table 4).

Irrespective of the geographical origin, we could consistently identify the two most abundant MytM types: a consensus very similar to the original sequence (MytM_1, MGC05878) and a second one (MytM_2) similar to the sequence MytM-P recently described [39]. Jointly, MytM_1 and MytM_2 represent the 87, 78 and 76% of the MytM reads in the samples Pa, Vi and Ve, respectively (Vi MytM display higher sequence variability than the other two samples).

**Figure 4:**
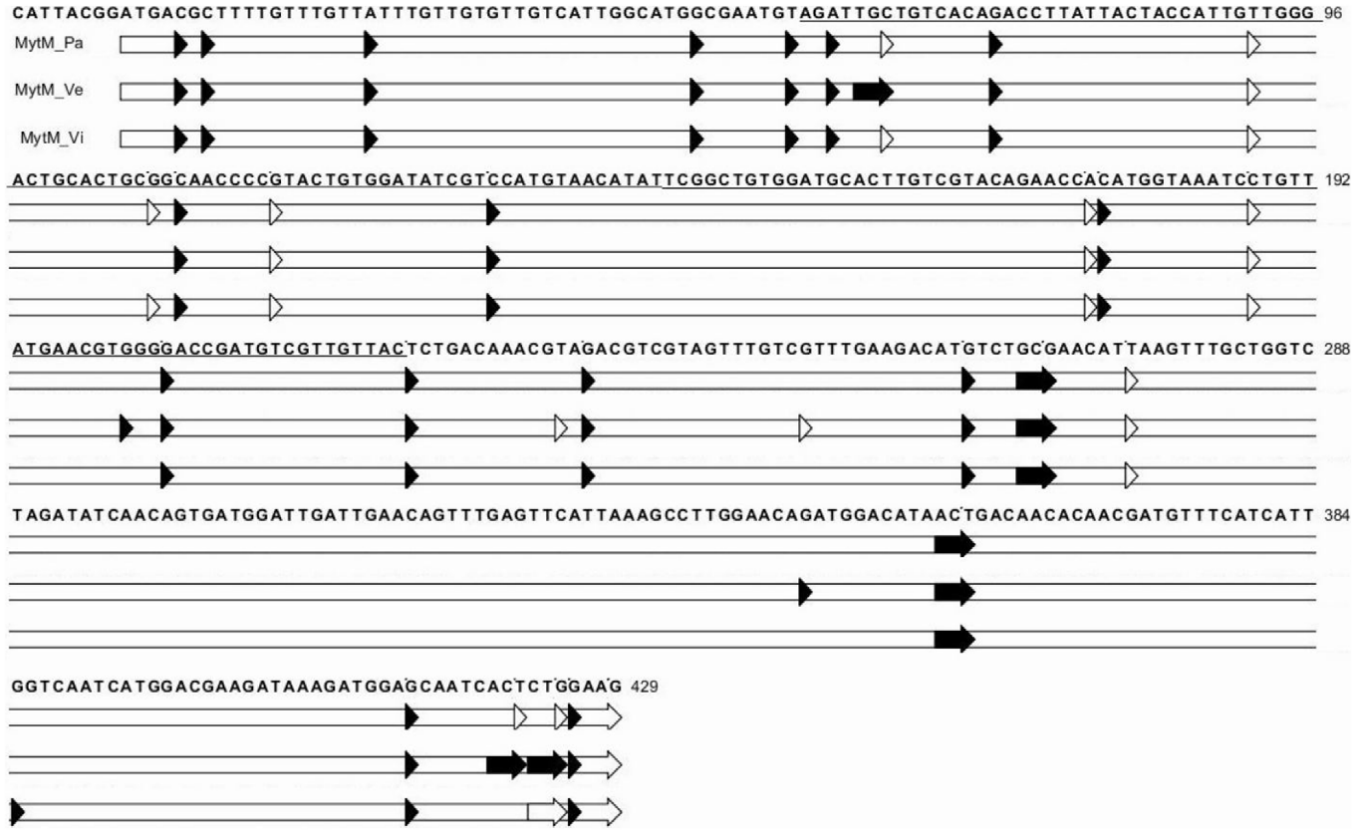
SNCs mapped along the MytM sequence in the Pa, Ve and Vi samples. Synonymous (empty) and non-synonymous (filled) changes are located on the AMP precursor sequence. Empty horizontal bars indicates cds, mature peptide sequence is underlined.

MytM_2 represents 20, 8 and 11% of the MytM reads in samples Pa, Vi and Ve, respectively, and displays 25 SNCs. Eighteen out of 25 changes are non-synonymous (7 in the signal peptide, 8 in the mature peptide and 3 in the C-terminal extension) and confirm the substitutions detected in MytM-P by Sonthi *et al.* using a Sanger approach. Figure 4 locates all genuine SNCs along the original MytM sequence (cds).

Partial gene sequences including the cds have been reported for MytC and denote 3 exons and 2 introns, with the mature peptide entirely located in exon 2 [29], [36].

The MytC pyrosequencing yielded 22,119 aligned reads fully covering the cds (71%, 88% and 79% of them differing from the original sequence in the Pa, Vi and Ve samples, respectively). The Pa, Vi and Ve MytC reads could be grouped in 844, 777 and 468 clusters or singletons (823, 752 and 466 of high quality), respectively. In total, 18113 high quality reads (82%) were translated in amino acids and produced 41–93 expected peptide variants (Table 4) without evidence of prevailing variation patterns.

**Figure 5:**
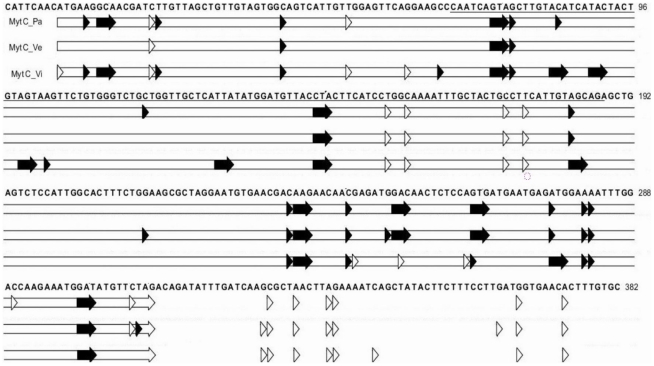
SNCs mapped along the MytC sequence in the Pa, Ve and Vi samples. Synonymous (empty) and non-synonymous (filled) changes are located on the AMP precursor sequence. Empty horizontal bars indicates cds, mature peptide sequence is underlined.

Despite the remarkable number of sequence variants, 98.8% of the MytC peptide clusters retained the typical cysteine array. Figure 5 locates all genuine SNCs along the original sequence of MytC. Amplicon pyrosequencing confirmed 3/5, 17/27 and 19/33 changes previously detected in the signal mature peptide, and C-terminal regions, respectively [36] and revealed additional changeable positions. As observed in MytM, different SNC combinations increase the total number of possible peptide variants.

The maps locating all synonymous and non-synonymous SNCs along the transcript sequence (cds) of the remaining AMPs are reported in Supplementary Materials (File S5) for comparison.

## Discussion

This study intended to assess, by high-throughput amplicon sequencing, the natural variability of nine AMP precursor sequences found expressed in the Mediterranean mussel. For this purpose, we sampled mussels from farming sites subjected to common European regulations and sanitary controls in three producer countries.

Thirteen sequences almost covering the AMP cds (File S1) have been successfully amplified from hemocyte RNA samples representing mussels farmed in France, Spain and Italy, as well as native mussels of the Venice lagoon area, before and after Vibrio injection, for coupled comparison.

Sample preparation is a decisive step of the sequencing workflow, due to the difficulty in preparing a well-balanced unique amplicon pool for the emulsion PCR and subsequent pyrosequencing. For this purpose, we measured and equalized the concentrations of each amplicon with great attention before pooling. Although the two sequencing half plates produced similar read numbers per sample, a general variability of coverage depth between AMP amplicons was finally evident (Table 3, Figure 1). Nevertheless, we obtained at least 500× amplicon coverage with one only exception (MytM in the Ve ft sample).

Stringent criteria were then used to identify genuine SNCs, removing false positives without losing substantial information, and we could retain most of the output reads (82% and 86% to exemplify Myticin C and Mytimycin, respectively). At first look, the output reads of each AMP appeared very diverse, 88% with at least one SNC on average (100% for MGDt, no matter from which sample). The analysis of SNC frequency per base enabled us to rank the selected AMPs on the basis of the transcript variability (Figure 4). Despite the invariance of the cysteine array, each AMP showed typical levels of diversity irrespective of the geographical origin, with a majority of common SNCs present in all samples (86%, i.e. 134 SNCs) and the Vi sample showing the greatest number of SNCs (292 in total). Moreover, we did not see evidence of increased AMP sequence diversity in farmed and native mussels injected with a high dose of live *Vibrio* cells. Compared to the samples prepared from 100 mussels (Pa, Vi, Ve, Ve nc), those prepared from 40 mussels (Ve ft, Ve nt) showed fewer SNCs (−11%); a fact indicating that the sample size can limit the amount of detectable sequence variants.

The ratio of non-synonymous vs. synonymous changes (ω) substantiates the evolutionary diversification of the mussel AMP isotypes and suggests the functional advantage of transcript variability for most of the analyzed AMPs (ω values higher than 1, indicative of positively selected residues, were frequently detected). However, the ω values did not reflect precisely the classification based on SNC frequencies and some AMP (e.g. MytlD) may have been subjected to higher evolutionary pressure than others.

The virtual translation of the transcript consensuses resulting from read clustering allowed the identification of AMP isotypes with a relatively low number of SNCs and high sequence diversity according to different SNC combinations (e.g. MytB and MGDt). In the case of mytimycin, we confirmed two major sequence types previously described [39] with no evidence of additional variants. For the remaining AMPs, it was not possible to identify specific patterns of variation (amino acid changes combined together without scheme).

In conclusion, the sequence data reported in this study further emphasize the sequence diversity of mussel AMP precursors. Redundant expression of diverse AMPs with a broad range of action could be regarded as a strategy to reinforce the host response against invaders (foes trying in their turn to escape detection and the host reactions) while the immune system also has to maintain the organism homeostasis with appropriate responses to commensal microbes (friends) and to danger signals released by damaged host cells [54]. On the other hand, environmental factors act as selective force only if they change the distribution of host genotypes (affecting only some genotypes, not all), thus influencing the immune system evolution of the host in the context of its life-history and population traits [55].

The isotype diversity levels found in this study might result from events occurring at DNA level as well as post-transcriptional changes such as deaminase-mediated cytidine to uridine transitions [14], [56]–[57]. Hence, targeted sequence enrichment and extension strategies applied to genomic DNA could identify active and remnant gene copies of each AMP isotype, and reveal the mechanisms underlying the observed sequence variation.

## Materials and Methods

### Sampling sites, treatment and RNA extraction

Adult mussels (*Mytilus galloprovincialis*) with a shell length of 6–8 cm and mixed sex were obtained from commercial shellfish stocks near Palavas (Pa, Mediterranean Sea, France; 43°31′49 N, 03°54′53 E), Ria de Vigo (Vi, Atlantic Ocean, Spain; 42°14′32 N, 08°48′26 E) and off-shore Venice (Ve, North Adriatic Sea, Italy; 45°18′29.8 N, 12°21′32.0 E). In addition, we collected wild mussels from the industrial canals of Porto Marghera (Lagoon of Venice, Italy; 45°27′33.5 N, 12°15′41 E). More than 100 animals per group were sampled.

According to the EU Directive 91/492, mussels cultivated in waters classified A (e.g. ¼ mile off-shore in the Adriatic Sea) can be marketed without depuration and are assumed not to contain potential pathogens nor biotoxins. Due to heavy mixed pollution, shellfishing was prohibited since 1996 in the area from the industrial district (P. Marghera) to the Venice town, though the overall shellfish quality can be improved by 2 month-depuration in type A waters. Mussels farmed offshore or living in the industrial canals (Venice lagoon area) were acclimatized for one week in sea water collected at flood tide (32‰, 22°C) and fed with *Isochrisis galbana*. Following shell notching, 0.1 ml of exponentially growing bacteria (10^7^ *V. splendidus LGP32* cells) were injected into the posterior adductor muscle (samples Ve ft and Ve nt).

Hemolymph (1 ml per animal) was withdrawn from the posterior adductor muscle with a syringe containing 0.2 ml of Alsever solution (27 mM sodium citrate, 2.6 mM citric acid, 114 mM glucose and 72 mM NaCl in distilled water) adjusted at pH 7.4, and used to compose pools, each representative of 10 animals.

Haemocytes were pelleted by 15 min centrifugation at 800 xg (4°C), carefully resuspended in 200 µl of TRIZOL reagent (Invitrogen, Carlsbad, USA) and stored at −80°C until use. Total RNA was isolated according to the manufacturer's instructions and resuspended in RNAse-free water. A further purification step with LiCl 2 M was applied to remove possible contaminants. RNA concentration was measured by UV-spectrometry (ND1000, NanoDrop Technologies, Wilmington, USA) and the RNA integrity was verified by microcapillary electrophoresis (RNA 6000 Nano LabChip, Agilent Technologies, Palo Alto, USA).

Finally, equal quantities of each RNA pool (N = 10) were mixed together to compose a unique pool per sample (N = 100 mussels for samples Pa, Vi, Ve and Ve nc; N = 40 for samples Ve ft and Ve nt).

### cDNA

cDNA was synthesized from 1 µg of total RNA using SuperScript II enzyme and oligo(dT)_18_ primers (Invitrogen), following the manufacturer's instruction. To increase cDNA yield, the reaction was extended for a second hour, adding 0.5 µl of enzyme. cDNA was then purified with MinElute PCR Purification Kit (Qiagen, Hilden, Germany).

### Primer design

Primer design was performed on the raw EST sequences denoting 9 mussel AMP isoforms in Mytibase (http://mussel.cribi.unipd.it).

All available ESTs for each selected AMP precursor were aligned using ClustalW [58] and primers were designed on conserved regions flanking the cds, whenever possible, with Primer3 [59]. Due to the pyrosequencing limit of about 250 bp, read length in forward and reverse direction, the maximum length of the PCR products was set at 440 bp (File S1). Degenerated primers have been designed to consider the whole ESTs variability (MytlC, MGD1 and MGDt). Two partially overlapping amplicons were designed for longer cds (MytC, MytlB, and MytlC) or in cases where high sequence variability made the definition of a single primer pair difficult (MytA). Thereby, 13 amplicons were designed in total. Amplicon's specificity was tested using the BlastX algorithm [60].

A tagged sequencing strategy with 5′nucleotide barcodes was implemented to facilitate the parallel processing of multiple samples [61]–[62]. Briefly, the forward and reverse PCR primers were modified by 5′-addition of 39 unique 5-mer barcodes (File S2). Barcodes enable the identification of the 454 reads corresponding to specific AMP, amplicon and sample so that PCR amplicons derived from multiple reactions could be combined for the sequencing run. To reduce the likelihood of misidentification, barcodes were designed not to contain homopolymers and to differ each one by at least two bases according to Roche Life Science protocols. Finally, 19-mer sequences corresponding to either the 454 Roche A Adaptor (for forward primers) or B Adaptor (for reverse primers) were fused to each PCR primer (Fusion primer).

Thermodynamic properties of Fusion primers were controlled to avoid the formation of hetero- or homo-dymer (OligoAnalyzer 3.1, http://eu.idtdna.com). Melting temperatures were fixed according to Primer3 software [63], adding 5 (±1) °C following HF Phusion polymerase instructions (Finnzymes, Espoo, Finland).

Fusion primers were designed in two sets of 39 (13 amplicons×3 samples), with each primer pair having a unique barcode.

### PCR amplification

The PCR amplifications of 78 amplicons were carried out individually in a PCR volume of 50 µl with 20 ng of cDNA template, 1× Phusion HF buffer, 0.2 mM dNTPs, 1 U HF Phusion DNA polymerase, 1.5 µl DMSO and 0.2 µM of both forward and reverse primers. Amplification was performed in a Mastercycler Gradient Thermal Cycler (Eppendorf, Hamburg, Germany) programmed as follows: 98°C for 30 s followed by 35 cycles of 98°C for 10 s, 60–65°C for 20 s, 72°C for 30 s and a final extension step at 72°C for 5 min.

The resulting amplification products were run on a 2*%* agarose gel and visualized by SYBR Gold staining (Invitrogen) using UV light transillumination (Gel Doc XR System, Bio-Rad, Hercules, USA). Unspecific small products and primer–dimers were removed using the Agencourt AMPure system (AmPure PCR Purification kit, Brea, USA) and amplicons integrity was confirmed with Agilent 2100 Bioanalyzer (DNA-1000 chip). Good quality amplicons were finally used to compose an equimolecular pool; the number of molecules of each amplicon was calculated with the following formula:
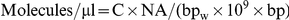

C: sample concentration (ng/µl)
N_A_: Avogadro constant
bp_w_: average pair basis weight (g)
*bp*: pair basis number

Massively parallel 454 pyrosequencing (FLX-System, Roche Life Sciences, Branford, USA) was performed by BMR-Genomics (www.bmr-genomics.it) using two PicoTiter half plates. Reads have been recorded at the Sequence Read Archive accessible at http://www.ncbi.nlm.nih.gov/Traces/sra (submission ID: SRA038518.3). Six 454-output files corresponding to the 6 sequenced samples are available (SRR286638.1, SRR286639.1, SRR286640.1, SRR286641.1, SRR287657.1 and SRR287658.1).

### Data analysis

Tag sequence were used as keys to part the unprocessed 454 reads into the 6 different samples by means of the GS Amplicon Variant Analyzer Software (AVAST, Roche Life Sciences). Reads in raw format were trimmed using quality score (limit 0.05) and minimum length equal to 100 bp. Subsequently, the output reads were aligned to a backbone consisting of the 9 AMP original transcripts, as obtained from the 13 reference sequences, with CLC Genomic Workbench version 4.6 (CLC Bio, Katrinrbjerg, Denmark). The total number of sequenced bases divided by the length of the amplified transcript provided the average base coverage per AMP.

The reads of each mapping (AMP isotype) showing the same length and 100% similarity were clustered together. Single nucleotide changes (SNCs) were detected considering all the aligned reads of each mapping. Non-specific and low quality matches are ignored during the process and SNCs were considered genuine only when covered at least 30×, with a minimum frequency of 3%, and setting the quality level of the changed base and surrounding bases to at least 20 and 15, respectively. SNCs located in the same codon were merged. The expected amino acid changes in the precursor and cds peptide sequences were deduced by virtual translation, and the ratio (ω) between non-synonymous and synonymous changes was also computed. SNC frequency per base in the cds region was calculated for each AMP and sample with the following formula:
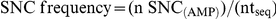

nt_seq_: cds sequenced nucleotides
n SNC_(AMP)_: number of genuine SNC per amplicon in each sample

To assess possible differences in levels of sequence diversity between the AMP precursors amplified from each of the 6 samples, data were analysed with 1-way ANOVA (α = 0.001). The null hypothesis predicted that all AMPs had the same variation rate in all samples. If not, Tukey's Honestly Significant Difference test (HSD, α = 0.05) could then discriminate different AMP groups. Genuine SNCs were mapped on the 9 AMP sequences using CLC Genomic Workbench. The cds, signal peptide, mature peptide with the cysteine array and C-terminal regions were systematically localized.

Once the correct sequence reading frame was defined the sequences covered at least 3× were virtually translated to investigate the overall patterns of variation for each AMP transcript precursor in the Pa, Vi and Ve samples. Leftover low quality read ends were manually trimmed and redundancy was removed by using Jalview [64].

## Supporting Information

File S1 **Number of reads per AMP and sample.**
(XLS)

File S2 **Detailed list of all SNCs detected per AMP precursor in the 6 samples, with type of change and related frequency.**
(XLS)

File S3 **The maps locating all synonymous and non-synonymous SNCs along the transcript sequence (cds) of the AMPs.**
(TIF)

File S4 **The 13 reference sequences designed to cover 9 AMP precursor transcripts of** ***M. galloprovincialis*** **.** Sequence number and expected amplicon length with related transcript ID, precursor length, and number of clustered ESTs. All reference sequences are also reported in detail.
(DOC)

File S5 **Fusion primer pairs used for the AMP amplification, primer length, melting temperature and GC percentage.**
(XLS)
