# Supplementary figures and images for: Massively Parallel Amplicon Sequencing Reveals Isotype-Specific Variability of Antimicrobial Peptide Transcripts in Mytilus galloprovincialis
Source: PLoS One. 2011 Nov 7;6(11):e26680. doi: 10.1371/journal.pone.0026680 (PMC3210125; doi:10.1371/journal.pone.0026680)

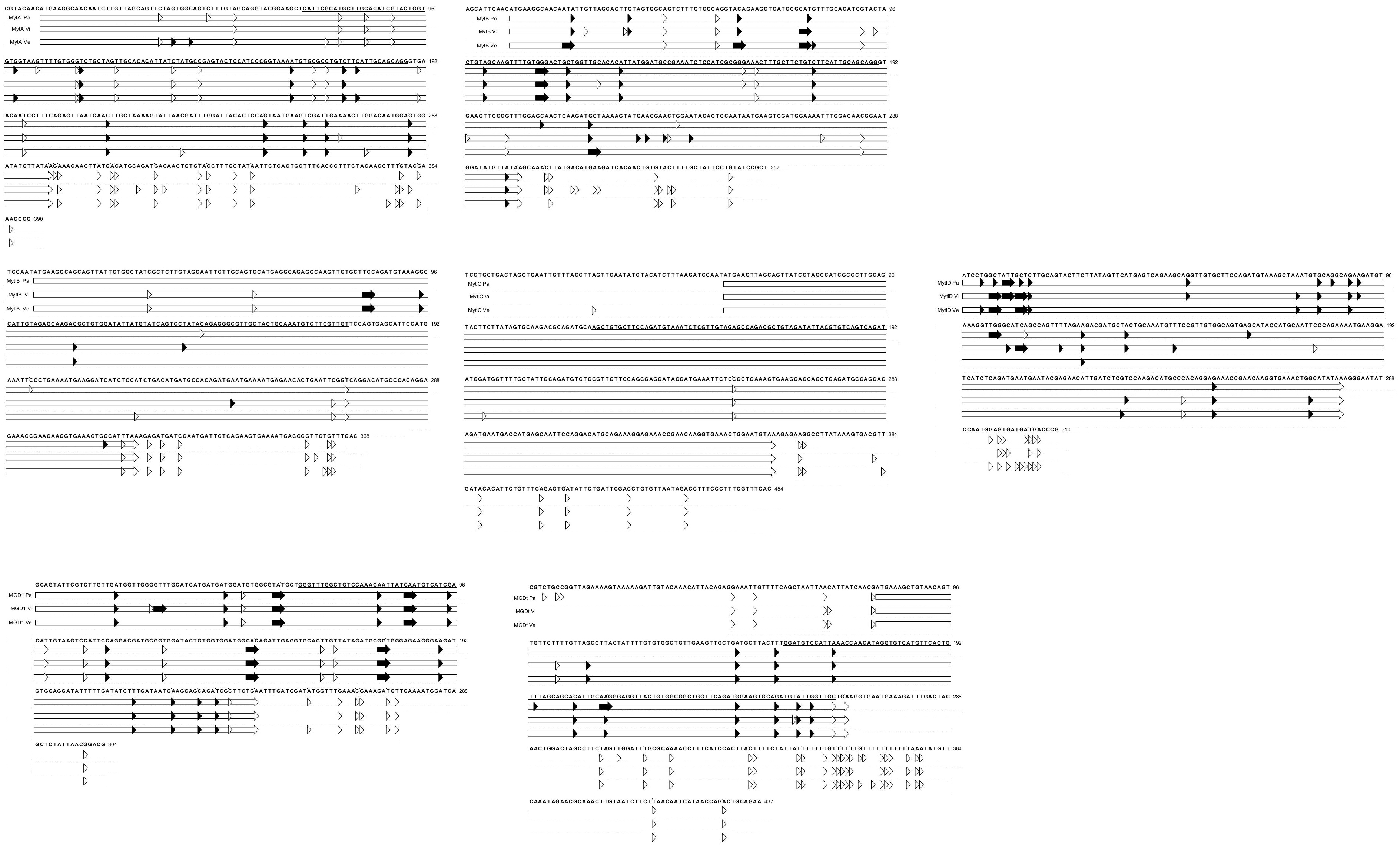

Supplement: File S3 — The maps locating all synonymous and non-synonymous SNCs along the transcript sequence (cds) of the AMPs. (TIF) [file pone.0026680.s003.tif]
