# Supplementary material for: Massively Parallel Amplicon Sequencing Reveals Isotype-Specific Variability of Antimicrobial Peptide Transcripts in Mytilus galloprovincialis
Source: PLoS One. 2011 Nov 7;6(11):e26680. doi: 10.1371/journal.pone.0026680 (PMC3210125; doi:10.1371/journal.pone.0026680)
Supplement: File S4 — The 13 reference sequences designed to cover 9 AMP precursor transcripts of M. galloprovincialis . Sequence number and expected amplicon length with related transcript ID, precursor length, and number of clustered ESTs. All reference sequences are also reported in detail. (DOC) [file pone.0026680.s004.doc]

**File S1.** The 13 reference sequences designed to cover 9 AMP precursor transcripts of *M. galloprovincialis.* Sequence number and expected amplicon length with related transcript ID, precursor length, and number of clustered ESTs. All reference sequences are also reported in detail.

| **Reference sequence** | **Amplicon lenght (nt)** | **AMP** | **Precursor lenght (nt)** | **Mytibase transcript ID & clustered ESTs** | | | |
| --- | --- | --- | --- | --- | --- | --- | --- |
| 1, 2 | 356, 390 | MytA | 288 | MGC00270 |  | 70 |  |
|  |  |  |  | MGC00087 |  | 24 |  |
|  |  |  |  | MGC00421 |  | 5 |  |
|  |  |  |  | MGC01070 |  | 1 |  |
|  |  |  |  | MGC01140 |  | 1 |  |
| 3 | 357 | MytB | 288 | MGC00422 |  | 60 |  |
|  |  |  |  | MGC00840 |  | 1 |  |
|  |  |  |  | MGC03686 |  | 1 |  |
| 4, 5 | 229, 279 | MytC | 300 | MGC00300 |  | 145 |  |
|  |  |  |  | MGC01021 |  | 2 |  |
| 6, 7 | 305, 203 | MytlB | 309 | MGC00227 |  | 126 |  |
|  |  |  |  | MGC00423 |  | 15 |  |
|  |  |  |  | MGC00659 |  | 12 |  |
| 8, 9 | 320, 370 | MytlC | 300 | MGC00294 |  | 135 |  |
|  |  |  |  | MGC00926 |  | 3 |  |
| 10 | 310 | MytlD | 291 | MGC00317 |  | 16 |  |
| 11 | 429 | MytM | 456 | MGC05878 |  | 1 |  |
|  |  |  |  | MGC03823 |  | 4 |  |
| 12 | 304 | MGD1 | 246 | MGC00280 |  | 18 |  |
|  |  |  |  | MGC00431 |  | 5 |  |
|  |  |  |  | MGC00451 |  | 2 |  |
| 13 | 437 | MGDt | 183 | MGC00358 |  | 17 |  |

**Reference 1 MytA**

GGCAACAATCTTGTTAGCAGTTCTAGTGGCAGTCTTTGTCGCAGGTACGGAAGCTCATTCGCACGCTTGTACATCATACTGGTGTGGTAAGTTTTGTGGAACTGCTAGTTGCACACATTATTTATGCAGAGTACTCCATCCCGGTAAACTGTGTGTATGTGTTCATTGCAGCAGGGTGAACAATCCTTTCAGAGTTAATCAAGTTGCTAAAAGTATTAACGATTTGGATTACACTCCAATAATGAAGTCGATGGAAAACTTGGACAATGGAATGGATATGTTATAAGCAAACAACTTATGCAATGAAGATCACAACTGTGAACCTTTGCTATCATTCTCACTGCTTTTCACCTTTC

**Reference 2 MytA**

CGTACAACATGAAGGCAACAATCTTGTTAGCAGTTCTAGTGGCAGTCTTTGTAGCAGGTACGGAAGCTCATTCGCATGCTTGCACATCGTACTGGTGTGGTAAGTTTTGTGGGTCTGCTAGTTGCACACATTATCTATGCCGAGTACTCCATCCCGGTAAAATGTGCGCCTGTCTTCATTGCAGCAGGGTGAACAATCCTTTCAGAGTTAATCAACTTGCTAAAAGTATTAACGATTTGGATTACACTCCAGTAATGAAGTCGATTGAAAACTTGGACAATGGAGTGGATATGTTATAAGAAACAACTTATGACATGCAGATGACAACTGTGTACCTTTGCTATAATTCTCACTGCTTTCACCCTTTCTACAACCTTTGTACGAAACCCG

**Reference 3 MytB**

AGCATTCAACATGAAGGCAACAATATTGTTAGCAGTTGTAGTGGCAGTCTTTGTCGCAGGTACAGAAGCTCATCCGCATGTTTGCACATCGTACTACTGTAGCAAGTTTTGTGGGACTGCTGGTTGCACACATTATGGATGCCGAAATCTCCATCGCGGGAAACTTTGCTTCTGTCTTCATTGCAGCAGGGTGAAGTTCCCGTTTGGAGCAACTCAAGATGCTAAAAGTATGAACGAACTGGAATACACTCCAATAATGAAGTCGATGGAAAATTTGGACAACGGAATGGATATGTTATAAGCAAACTTATGACATGAAGATCACAACTGTGTACTTTTGCTATTCCTGTATCCGCT

**Reference 4 MytC**

CATTCAACATGAAGGCAACGATCTTGTTAGCTGTTGTAGTGGCAGTCATTGTTGGAGTTCAGGAAGCCCAATCAGTAGCTTGTACATCATACTACTGTAGTAAGTTCTGTGGGTCTGCTGGTTGCTCATTATATGGATGTTACCTACTTCATCCTGGCAAAATTTGCTACTGCCTTCATTGTAGCAGAGCTGAGTCTCCATTGGCACTTTCTGGAAGCGCTAGGAATGT

**Reference 5 MytC**

GTTCTGTGGGTCTGCTGGTTGCTCATTATATGGATGTTACCTACTTCATCCTGGCAAAATTTGCTACTGCCTTCATTGTAGCAGAGCTGAGTCTCCATTGGCACTTTCTGGAAGCGCTAGGAATGTGAACGACAAGAACAACGAGATGGACAACTCTCCAGTGATGAATGAGATGGAAAATTTGGACCAAGAAATGGATATGTTCTAGACAGATATTTGATCAAGCGCTAACTTAGAAAATCAGCTATACTTCTTTCCTTGATGGTGAACACTTTGTGC

**Reference 6 MytlB**

TCCAATATGAAGGCAGCAGTTATTCTGGCTATCGCTCTTGTAGCAATTCTTGCAGTCCATGAGGCAGAGGCAAGTTGTGCTTCCAGATGTAAAGGCCATTGTAGAGCAAGACGCTGTGGATATTATGTATCAGTCCTATACAGAGGGCGTTGCTACTGCAAATGTCTTCGTTGTTCCAGTGAGCATTCCATGAAATTCCCTGAAAATGAAGGATCATCTCCATCTGACATGATGCCACAGATGAATGAAAATGAGAACACTGAATTCGGTCAGGACATGCCCACAGGAGAAACCGAACAAGGTGA

**Reference 7 MytlB**

CTTCGTTGTTCCAGTGAGCATTCCATGAAATTCCCTGAAAATGAAGGATCATCTCCATCTGACATGATGCCACAGATGAATGAAAATGAGAACACTGAATTCGGTCAGGACATGCCCACAGGAGAAACCGAACAAGGTGAAACTGGCATTTAAAGAGATGATCCAATGATTCTCAGAAGTGAAAATGACCCGTTCTGTTTGAC

**Reference 8 MytlC**

TCCTGCTGACTAGCTGAATTGTTTACCTTAGTTCAATATCTACATCTTTAAGATCCAATATGAAGTTAGCAGTTATCCTAGCCATCGCCCTTGCAGTACTTCTTATAGTGCAAGACGCAGATGCAAGCTGTGCTTCCAGATGTAAATCTCGTTGTAGAGCCAGACGCTGTAGATATTACGTGTCAGTCAGATATGGATGGTTTTGCTATTGCAGATGTCTCCGTTGTTCCAGCGAGCATACCATGAAATTCTCCCCTGAAAGTGAAGGACCAGCTGAGATGCCAGCACAGATGAATGACCATGAGCAATTCCAGGAC

**Reference 9 MytlC**

TCGCCCTTGCAGTACTTCTTATAGTGCAAGACGCAGATGCAAGCTGTGCTTCCAGATGTAAATCTCGTTGTAGAGCCAGACGCTGTAGATATTACGTGTCAGTCAGATATGGATGGTTTTGCTATTGCAGATGTCTCCGTTGTTCCAGCGAGCATACCATGAAATTCTCCCCTGAAAGTGAAGGACCAGCTGAGATGCCAGCACAGATGAATGACCATGAGCAATTCCAGGACATGCAGAAAGGAGAAACCGAACAAGGTGAAACTGGAATGTAAAGAGAAGGCCTTATAAAGTGACGTTGATACACATTCTGTTTCAGAGTGATATTCTGATTCGACCTGTGTTAATAGACCTTTCCCTTTCGTTTCAC

**Reference 10 MytlD**

ATCCTGGCTATTGCTCTTGCAGTACTTCTTATAGTTCATGAGTCAGAAGCAGGTTGTGCTTCCAGATGTAAAGCTAAATGTGCAGGCAGAAGATGTAAAGGTTGGGCATCAGCCAGTTTTAGAAGACGATGCTACTGCAAATGTTTCCGTTGTGGCAGTGAGCATACCATGCAATTCCCAGAAAATGAAGGATCATCTCAGATGAATGAATACGAGAACATTGATCTCGTCCAAGACATGCCCACAGGAGAAACCGAACAAGGTGAAACTGGCATATAAAGGGAATATCCAATGGAGTGATGATGACCCG

**Reference 11 MytM**

CATTACGGATGACGCTTTTGTTTGTTATTTGTTGTGTTGTCATTGGCATGGCGAATGTAGATTGCTGTCACAGACCTTATTACTACCATTGTTGGGACTGCACTGCGGCAACCCCGTACTGTGGATATCGTCCATGTAACATATTCGGCTGTGGATGCACTTGTCGTACAGAACCACATGGTAAATCCTGTTATGAACGTGGGGACCGATGTCGTTGTTACTCTGACAAACGTAGACGTCGTAGTTTGTCGTTTGAAGACATGTCTGCGAACATTAAGTTTGCTGGTCTAGATATCAACAGTGATGGATTGATTGAACAGTTTGAGTTCATTAAAGCCTTGGAACAGATGGACATAACTGACAACACAACGATGTTTCATCATTGGTCAATCATGGACGAAGATAAAGATGGAGCAATCACTCTGGAAG

**Reference 12 MGD1**

GCAGTATTCGTCTTGTTGATGGTTGGGGTTTGCATCATGATGATGGATGTGGCGTATGCTGGGTTTGGCTGTCCAAACAATTATCAATGTCATCGACATTGTAAGTCCATTCCAGGACGATGCGGTGGATACTGTGGTGGATGGCACAGATTGAGGTGCACTTGTTATAGATGCGGTGGGAGAAGGGAAGATGTGGAGGATATTTTTGATATCTTTGATAATGAAGCAGCAGATCGCTTCTGAATTTGATGGATATGGTTTGAAACGAAAGATGTTGAAAATGGATCAGCTCTATTAACGGACG

**Reference 13 MGDt**

CGTCTGCCGGTTAGAAAAGTAAAAAGATTGTACAAACATTACAGAGGAAATTGTTTTCAGCTAATTAACATTATCAACGATGAAAGCTGTAACAGTTGTTCTTTTGTTAGCCTTACTATTTTGTGTGGCTGTTGAAGTTGCTGATGCTTACTTTGGATGTCCATTAAACCAACATAGGTGTCATGTTCACTGTTTAGCAGCACATTGCAAGGGAGGTTACTGTGGCGGCTGGTTCAGATGGAAGTGCAGATGTATTGGTTGCTGAAGGTGAATGAAAGATTTGACTACAACTGGACTAGCCTTCTAGTTGGATTTGCGCAAAACCTTTCATCCACTTACTTTTCTATTATTTTTTTGTTTTTTGTTTTTTTTTTTAAATATGTTCAAATAGAACGCAAACTTGTAATCTTCTTAACAATCATAACCAGACTGCAGAA
